# The Antidepressant Sertraline Modulates Gene Expression and Alternative Splicing Events in the Dermatophyte *Trichophyton rubrum*: A Comprehensive Analysis

**DOI:** 10.3390/genes16020146

**Published:** 2025-01-24

**Authors:** Carlos H. Lopes Rocha, Flaviane M. Galvão Rocha, Pablo R. Sanches, Antonio Rossi, Nilce M. Martinez-Rossi

**Affiliations:** Department of Genetics, Ribeirão Preto Medical School, University of São Paulo, Ribeirão Preto 14049-900, SP, Brazil; henriqueclr16@alumni.usp.br (C.H.L.R.); flavianerocha@usp.br (F.M.G.R.); psanches@usp.br (P.R.S.); anrossi@usp.br (A.R.)

**Keywords:** dermatophytosis, sertraline, alternative splicing, intron retention, kinase, pathogenic fungi

## Abstract

Background/Objectives: Dermatophytosis, a prevalent fungal infection of keratinized tissues, is primarily caused by the filamentous fungus *Trichophyton rubrum*. Sertraline (SRT), an antidepressant with antifungal activity, has already demonstrated therapeutic potential against this fungus. Elucidating the effects of SRT may provide insights into its mechanism of action and fungal adaptation to this drug. Differential gene expression and alternative splicing (AS) facilitate fungal adaptations to various environmental conditions. This study aimed to provide a comprehensive overview of AS events and their implications in *T. rubrum* cultivated under sub-inhibitory concentrations of SRT. Method: The transcriptome of *T. rubrum* challenged with SRT was analyzed to detect AS events. Results: RNA-seq analysis revealed that SRT affected transcriptional and post-transcriptional events in numerous *T. rubrum* genes, including those encoding transcription factors, kinases, and efflux pumps. Among the AS events, intron retention was predominant. After 12 h of SRT exposure, intron-3 retention levels in the serine/arginine protein kinase mRNA transcripts were significantly increased compared with those in the control. This new isoform would produce a putative protein that partially lost its phosphotransferase domain. Conclusions: These findings highlight the potential mechanisms of action of SRT and suggest how *T. rubrum* adapts itself to this drug.

## 1. Introduction

The increased incidence of fungal infections in humans, particularly among immunocompromised individuals, is a major public health issue [1,2]. Dermatophytosis is among the most common fungal infections in keratinized tissues such as the skin and nails, and is primarily caused by *Trichophyton rubrum* [3,4].

Treating fungal diseases is generally protracted and expensive, with the risk of developing antifungal resistance [5], a critical evolutionary phenomenon in fungi to adapt to extreme conditions [5,6]. Moreover, although genetic mutations in fungi occur at a relatively low frequency, the selective pressure exerted by the continuous use of antifungal agents favors the emergence of resistant strains, which eventually dominate the population [3]. Furthermore, only a few drugs can combat these pathogens owing to the limited number of cellular targets [1,7].

Repurposing sertraline (SRT), an antidepressant with antifungal activity, is a promising therapeutic strategy for various fungal diseases [8,9,10,11,12]. Owing to its established safety profile and mechanisms of action, the use of SRT decreases the duration and cost associated with the development of novel therapeutics. SRT is effective as both a monotherapy and in combination with other antifungal agents [13,14,15,16,17,18].

The antifungal efficacy of SRT was first reported in 2001, indicating its potential as a therapeutic agent [19,20]. Specifically, three patients with premenstrual dysphoric disorder and recurrent vulvovaginal candidiasis were treated with SRT. Both symptoms were eliminated. Furthermore, SRT exhibited antifungal activity in vitro against *Candida albicans*, *C. glabrata*, and *C. tropicalis* [20]. These findings underscore the broad-spectrum antifungal properties of SRT [21]. In mammals, SRT blocks the 5-hydroxytryptamine transporter, impeding serotonin reuptake [22].

In yeast, SRT acts on the phospholipid membranes of acidic organelles [23]. Furthermore, SRT exerts antifungal effects against *Cryptococcus neoformans* by obstructing translation, thereby inhibiting protein synthesis [19]. SRT also impedes the transition of *C. auris* from yeast to the hyphal form, reducing biofilm formation by 71% and causing significant cellular membrane damage [24]. Understanding the intricate mechanisms of action of SRT in *T. rubrum* and the mechanism by which it reacts to different conditions is essential for its clinical use against dermatophytes.

Several factors contribute to *T. rubrum* adaptation, including alternative splicing (AS) [3], a post-transcriptional regulatory mechanism that generates different mRNAs from a single gene. AS is a complex phenomenon and enhances protein diversity and non-coding RNA production in response to factors such as host conditions, drug exposure, and nutrient availability [25]. This mechanism has been reported in various clinically important pathogens, such as *C. albicans* and *C. neoformans* [26,27]. Intron retention (IR) is the most prevalent AS event in *T. rubrum* and other fungi [28].

Protein kinases play crucial roles in fungi, serving as part of evolutionarily conserved adaptation mechanisms shared across species from yeast to humans [29]. Serine/arginine protein kinases (CMGC/SRPKs) represent a distinct protein cohort vital for post-translational modifications. CMGC kinases are essential for cellular signal transduction pathways and regulate cell cycle, growth, and specialization. SRPKs are involved in serine/arginine protein phosphorylation, a vital process governing constitutive splicing and AS in *C. albicans* and *Saccharomyces cerevisiae* [30].

In this study, we investigated the changes in global AS events in the transcripts of *T. rubrum* cultivated with sub-inhibitory SRT concentrations, with a particular example of the modulation of IR events in SRPK transcripts (*TERG_07061*).

## 2. Materials and Methods

### 2.1. Alternative Splicing Analysis

We analyzed RNA-seq data [31] from the Gene Expression Omnibus [32] database (accession number: GSE218521) to detect AS events in *T. rubrum* cultivated in SRT. The sequencing reads from the RNA-seq data were aligned to the *T. rubrum* reference genome using STAR aligners [33]. ASpli package was used to identify AS events using R software version 4.3.1 [34]. Differential expression analysis was performed using the Bioconductor DESeq package [35]. The Benjamin–Hochberg adjusted *p*-value was set to 0.05, and a cut-off of ±1.0 log2 fold change was used to assess the significant expression differences [36].

### 2.2. Strain and Growth Conditions

The *T. rubrum* strain CBS118892, was obtained from the Westerdijk Fungal Biodiversity Institute (Utrecht, The Netherlands) and originated from a patient with onychomycosis. This strain was grown on malt extract agar (Becton Dickinson, Franklin Lakes, NJ, USA) for 21 days at 28 °C to promote robust growth, facilitating spore production [31]. Conidial concentration was estimated using a Neubauer chamber with 0.9% NaCl. About 1 × 10^6^ conidia were added to 100 mL of liquid Sabouraud medium (Becton Dickinson, Franklin Lakes, NJ, USA), followed by pre-cultivation at 28 °C for 96 h under shaking. The mycelia obtained were transferred to 100 mL of liquid Sabouraud medium containing either a sublethal dose of SRT (70 mg/L; Cayman Chemical, Ann Arbor, MI, USA) or no drug (control) and incubated at 28 °C with shaking (120 rpm) for 3 and 12 h. The minimum inhibitory concentration of SRT for *T. rubrum* was determined following the recommendations of the Clinical and Laboratory Standards Institute, as previously reported [18].

### 2.3. Reverse Transcription–Quantitative Polymerase Chain Reaction (RT-qPCR) and Conventional PCR

The *TERG_07061* gene, which encodes a CMGC/SRPK protein kinase, exhibited IR3 events in RNA-seq assays at 3 and 12 h of SRT exposure. Gene expression was quantified by qPCR using the StepOnePlus Real-Time PCR System (Applied Biosystems, Waltham, MA, USA). Both qPCR and conventional PCR were performed using specific primer pairs designed with the Prime3Plus software (https://www.bioinformatics.nl/cgi-bin/primer3plus/primer3plus.cgi, accessed on 4 July 2022). The primers used for qPCR assays targeted specific regions of the transcripts, either including or excluding IR3, whereas those for conventional PCR assays were designed to flank the intronic region [37] (Supplementary Figure S1). All primer sequences are shown in Supplementary Table S1. Genes encoding glyceraldehyde-3-phosphate dehydrogenase and DNA-dependent RNA polymerase II served as endogenous controls for qPCR [31]. The thermocycler conditions for qPCR were as follows: an initial denaturation stage at 95 °C for 10 min, followed by 40 cycles of 95 °C for 15 s (denaturation) and 60 °C for 1 min (annealing and extension). Gene expression levels were calculated using the comparative 2^−∆∆CT^ method [18]. The products of conventional PCR were visualized on a 2% agarose gel under a transilluminator, as previously described [37].

### 2.4. In Silico Analysis of SRPK Isoforms

The *TERG_07061* gene sequence was identified using the Ensembl Fungi database (https://fungi.ensembl.org, accessed on 4 July 2022), using the coordinates for the retained intron (start: 209028; end: 209083). Using the BLAST tool [38], we searched for sequence similarity between the two SRPK isoforms (conventional and IR3). Then, the domains within the two isoforms were identified using the Interpro database [39]. To predict and align the three-dimensional models of the two isoforms, we used AlphaFold2 [40] and PyMOL [41], respectively. Subsequently, the Illustrator for Biological Sequences (IBS 1.0) was used to represent each isoform graphically [42].

### 2.5. Statistical Analyses

A paired Student’s *t*-test was used to compare the gene expression levels between the treated and control groups. The results are shown as the mean ± standard deviation of three independent biological replicates. One-way analysis of variance followed by Tukey’s post hoc test was used to compare the treatment and control groups. Statistical significance was set at *p* < 0.05. Prism v. 5.1 (GraphPad Software, San Diego, CA, USA) was used to generate graphs.

## 3. Results

**Figure 1:**
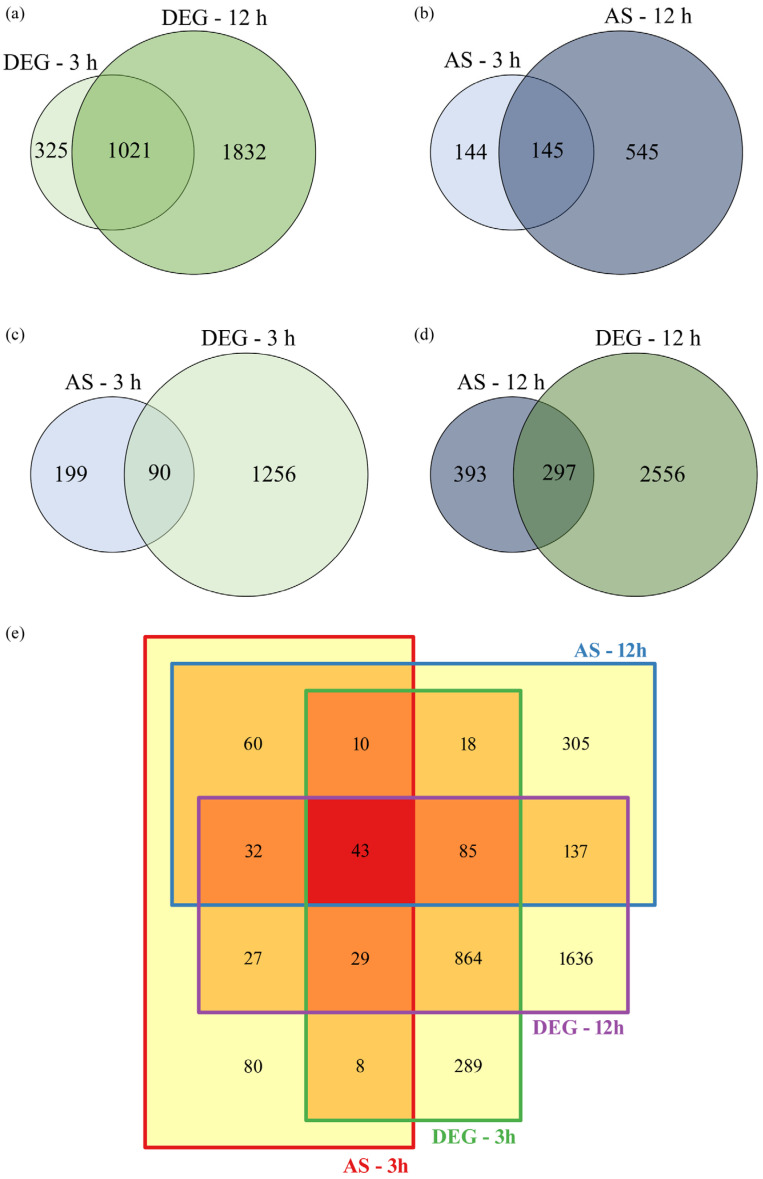
Venn diagrams illustrating the differentially expressed genes (DEGs) and the genes that underwent alternative splicing (AS) after *Trichophyton rubrum* exposure to sertraline for 3 and 12 h. (**a**) DEGs at 3 and 12 h. (**b**) AS at 3 and 12 h. Relationship between the number of genes undergoing AS and DEGs at 3 h (**c**) and 12 h (**d**). (**e**) Total DEGs and AS modulated at 3 and 12 h.

The *T. rubrum* transcriptome revealed 1346 and 2853 differentially expressed genes (DEG) after 3 and 12 h of SRT treatment, respectively (Figure 1a). Notably, 1021 genes exhibited differential expression independent of the culture time. Additionally, AS events were identified in 289 and 690 genes after 3 h and 12 h of SRT treatment, respectively. Of these, 145 genes did not show time dependence (Figure 1b).

Several genes exhibited differential expression and AS events simultaneously at 3 h (Figure 1c) and 12 h (Figure 1d) of SRT treatment. The 289 genes that underwent AS after 3 h of SRT treatment corresponded to approximately 21% of all DEGs (Figure 1c). This percentage increased to 24% after 12 h of treatment (Figure 1d). Transcriptome analysis also revealed 43 genes that were simultaneously modulated, exhibiting differential expression and AS events at 3 h and 12 h (Figure 1e).

A peculiar expression pattern was observed in these genes: the induction of differential expression downregulated AS events, and vice versa. This trend persisted in genes involved in multidrug resistance, and transcription factors, and one gene involved in eukaryotic translation initiation (Supplementary Table S2). Using the ASpli package, IR was identified as the most common AS event in *T. rubrum* cultivated in the presence of SRT. IR events primarily occurred at 12 h, with over 1000 detected events. At 3 h, nearly 100% of all AS events were of the IR type (Table 1).

We observed a large proportion of protein kinase-encoding genes among the differentially expressed genes in the RNA-seq analysis. At 3 h, 63 protein kinase-encoding genes were modulated, increasing to 132 at 12 h [31]. Therefore, we analyzed AS events in protein kinase-encoding transcripts. In total, 16 protein kinase-encoding transcripts underwent AS at 3 h and 41 underwent AS at 12 h in the SRT treatment, compared with the control without SRT. In addition, some genes exhibited more than one AS event (Supplementary Table S3).

**Figure 2:**
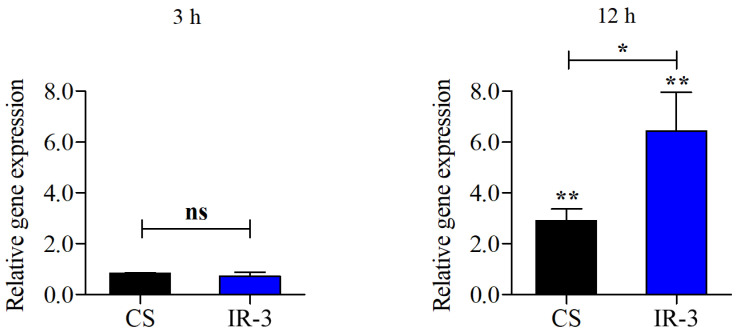
Quantitative polymerase chain reaction analysis of the expression levels of the conventional isoform (CS) and intron-3 retention (IR-3) in *T. rubrum* serine/arginine protein kinase (SRPK) gene (*TERG_07061*) after exposure to sertraline for 3 and 12 h. Asterisks above the bars indicate the significant differences compared with the control without SRT. Significant differences were evaluated using a *t*-test. ns, not significant; * *p* < 0.05 and ** *p* < 0.01 compared with the control.

*TERG_07061*, which encodes a CMGC/SRPK protein kinase, exhibited distinct behavior among the protein kinase-encoding genes. RNA-seq data revealed that this gene was repressed in response to SRT exposure. In contrast, IR3 was induced after 3 and 12 h of SRT treatment (Supplementary Table S2). Therefore, *TERG_07061* was selected for the subsequent validation of AS events, initially through RT-PCR (Supplementary Figure S1). The relative expression levels of the conventional isoform and the variant containing IR3 were quantified using RT-qPCR. The IR3 variant levels were significantly higher after 12 h of SRT exposure than in the control without SRT (Figure 2).

**Figure 3:**
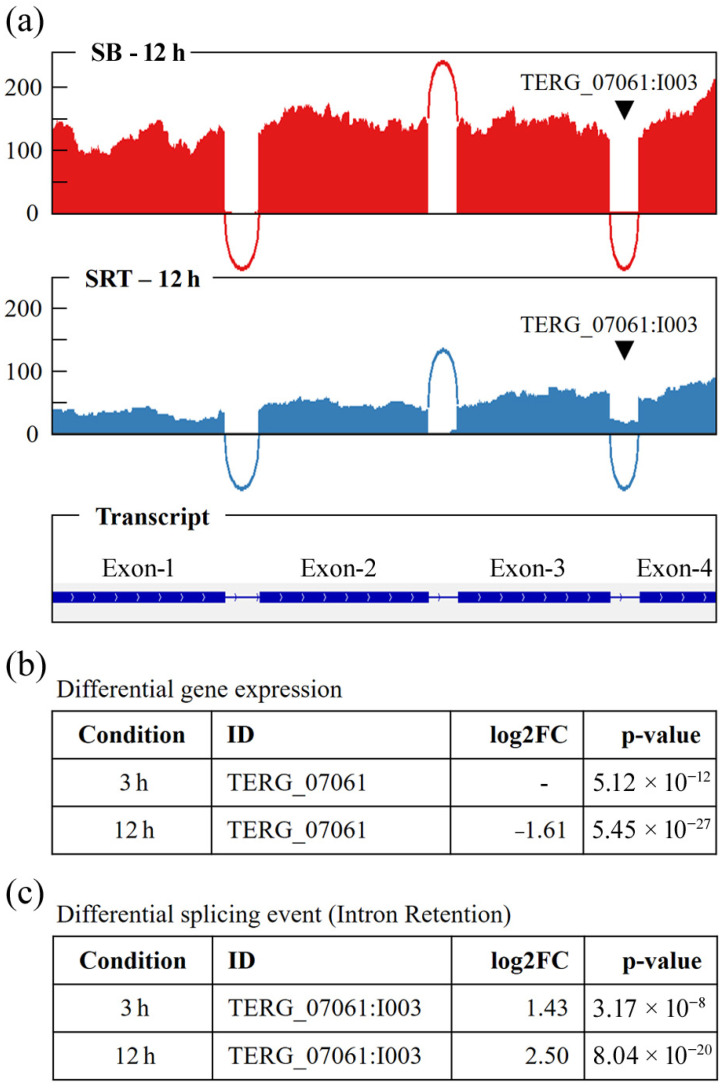
Sashimi plots of IR3 in *TERG_07061* gene. (**a**) Read depth indicating the quantity of reads generated during sequencing under the two tested conditions and at positions corresponding to the exons and introns of the *TERG_07061* transcripts. (**b**) Fold change (FC) and *p*-values of the *TERG_07061* DEG and (**c**) the alternative transcripts at 3 and 12 h. SB: control condition; SRT: sertraline treatment.

Figure 3 shows the Sashimi plots of IR3 in the *TERG_07061* gene, with the alignment of readings obtained from the RNA-seq analyses of the 12 h SRT treatment group, differential expression data, and IR3 events.

**Figure 4:**
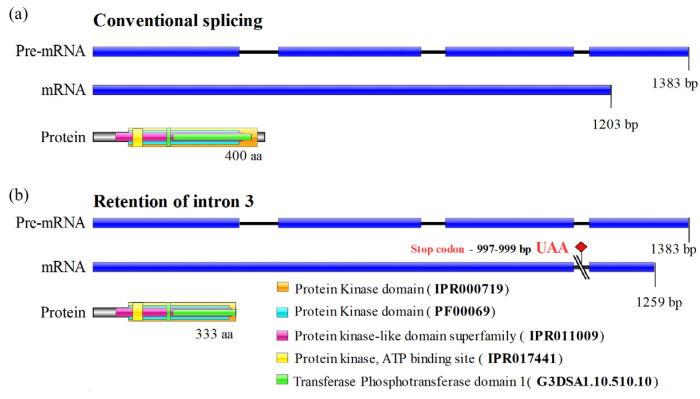
Graphical representation of splicing in *T. rubrum*. (**a**) Conventional splicing of SRPK (*TERG_07061*) and schematic representation of SRPK protein with its respective domains. (**b**) IR3 in the mRNA isoform of SRPK, and schematic representation of the SRPK protein with its respective domains. UAA, a stop codon, is indicated in red.

Usually, *TERG_07061* gene processing results in an mRNA of 1203 bp, yielding a single protein isoform with 400 amino acids. The pre-mRNA transcript contains three introns, as shown in Figure 4 and in the Ensembl Fungi database. When intron 3 was retained, a premature stop codon (UAA) was generated at bases 997–999 downstream of the start codon, resulting in an alternative isoform with 333 amino acids (Figure 4).

**Figure 5:**
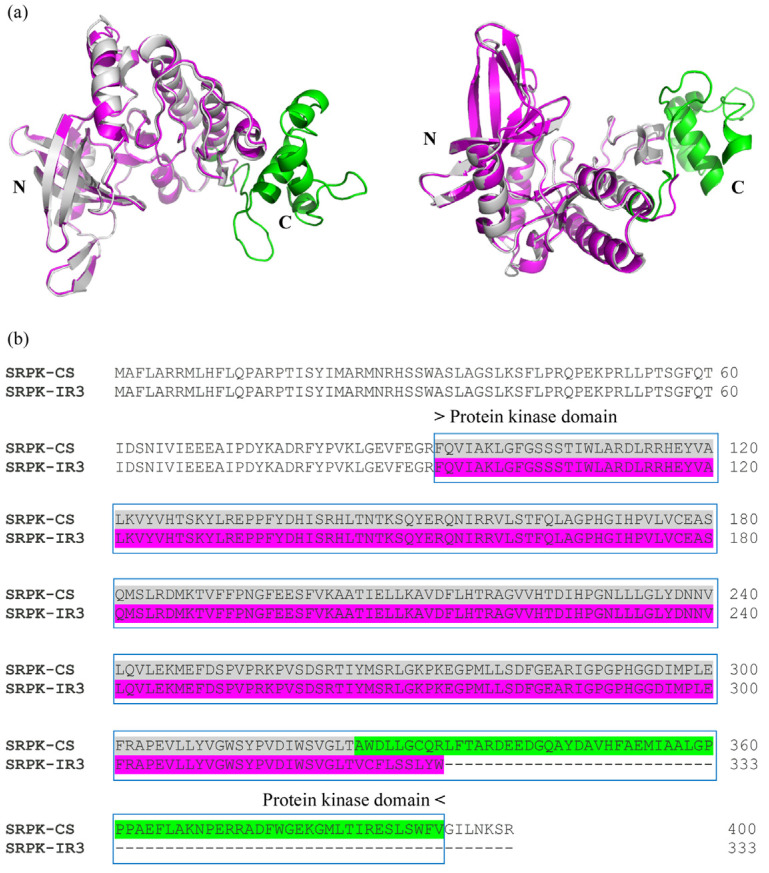
Superposition of the two isoforms of *T. rubrum* SRPK (*TERG_07061*). (**a**) Structural overlap between the catalytic domains of the conventional protein model (gray) obtained from the AlphaFold database (https://alphafold.ebi.ac.uk/search/text/TERG_07061, accessed on 4 July 2022) and the IR-3 protein model (magenta) predicted in this study using AlphaFold software. N: N-terminus; C: C-terminus of the protein. (**b**) Complete alignment of the sequences of the conventional protein (SRPK-CS) and the putative protein with IR3 (SRPK-IR3), indicating the catalytic domain regions.

Using InterPro, a database that facilitates functional protein analysis via classification into families and the prediction of domains and crucial sites, we found that the IR3 isoform preserved all CMGC/SRPK protein domains. However, alterations were observed in the transferase (phosphotransferase) domain, which lost part of its terminal region. Alignment of the protein kinase domains of both isoforms (conventional and IR3) indicated perfect identity up to the C-terminal region, where the amino acids of the IR3 isoform corresponded to part of intron 3 (Figure 5).

## 4. Discussion

AS events are associated with many biological processes, including development, drug resistance, adaptation to biotic and abiotic stress, response to pH changes, and virulence [37,43,44,45,46]. This study revealed IR as the most common AS event in *T. rubrum* challenged with SRT, which is consistent with previous reports, including those by our research group [25,47].

IR induces both adaptation and resistance to drugs by modulating gene expression, creating proteomic diversity, and facilitating rapid phenotypic changes. These responses enhance the organism’s ability to survive and thrive under drug-induced stress [26,48]. Some isoforms are active only under drug-induced stress, helping the organism adapt by using alternative metabolic pathways [47]. In this study, we observed the modulation of AS events in multidrug resistance and transcription factor transcripts, reflecting the intricate mechanisms developed by fungi to adapt to drug-induced stress, possibly conferring drug resistance.

This study also highlights the role of SRT exposure time in the incidence of AS events in *T. rubrum*. The longer the exposure time, the greater the number of genes exhibiting AS events, which may be related to fungal adaptation to SRT. These findings reveal the potential of targeting splicing events in drug therapy against fungi, paving the way for developing novel antifungal drugs.

The modulation of AS to overcome drug stress was previously identified in *C. albicans,* comparing azole-resistant and -sensitive isolates through RNA-Seq analysis [27]. In *S. cerevisiae*, IR governs cellular survival under starvation conditions, highlighting the significance of this mechanism in nutrient-deprived environments [49]. We previously demonstrated that the protein kinase encoded by the PAKA/STE20 gene, which plays a crucial role in mitogen-activated protein kinase signal transduction, retains intron 1 after exposure to undecanoic acid [37]. Moreover, we showed that the modulation of AS in genes encoding heat shock proteins facilitates adaptation to various stressors [44].

Different pre-mRNA isoforms may coexist in *T. rubrum*, and in the presence of SRT, the fungus modulates the expression of each isoform according to cellular requirements. We hypothesized that the induction of the SRPK IR3 isoform in *T. rubrum* in response to SRT is an adaptive strategy to counteract the repression of this gene caused by the drug. This repression likely triggers the activation of alternative pathways, such as the expression of non-canonical isoforms integrated into a regulatory network that maintains cellular equilibrium and homeostasis. Consequently, the selective production of isoforms underlies specific cellular functions in response to distinct stimuli [27,50,51].

However, the SRPK IR3 isoform encodes a putative kinase that loses part of the phosphotransferase domain, which probably alters its three-dimensional structure and affects its ability to interact with the substrate. Alternatively, the SRPK IR3 isoform may be involved in regulatory functions. For example, IR is essential for the mRNA accumulation of some genes in *C. neoformans* [26]. IR regulation could also be a strategy to adjust gene expression to environmental changes, supporting pathogen survival and proliferation under various conditions [26,52].

## 5. Conclusions

In summary, SRT affects both transcriptional and post-transcriptional events in *T. rubrum*. Our investigation revealed the modulation of AS events, particularly IR, in several genes of *T. rubrum* in response to SRT. This finding, along with the observation that the number of AS events increases with drug exposure time, and that some genes undergo both AS and expression modulation simultaneously, presents a promising area for further exploration. Notably, SRT does not induce these AS events directly; rather, they are modulated by its presence, likely as an adaptive mechanism. Our study confirmed the existence of a new isoform of the SRPK gene containing IR3, although there was no evidence that the putative protein would be functional. However, this isoform could assume distinct functionalities, such as gene regulation or altered kinase activity, compared with the conventional isoform. This discovery underscores the need for further research to better understand the potential implications of the isoforms revealed by RNA-seq analysis.

## Figures and Tables

**Table 1:** Alternative splicing events observed in response to sertraline.

Time	AS Genes	Intron Retention Events	Others AS Events	Total Events
3 h	289	349	2	351
12 h	690	1025	26	1051

*p* < 0.05 and |log2FC| ≥ 1.00 were considered statistically significant. AS: alternative splicing.

## Data Availability

The data presented in this study are available in this article and the accompanying Supplementary Data.

## Supplementary Materials

The following supporting information can be downloaded at: https://www.mdpi.com/article/10.3390/genes16020146/s1. Figure S1. Validation of alternative splicing event. Table S1. Primer sets used in conventional PCR and RT-qPCR assays. Table S2. List of 43 differentially expressed genes modulating alternative splicing events in response to sertraline. Table S3. Alternative splicing events in the pre-mRNA of kinase genes in response to sertraline.
