# Supplementary material for: The Antidepressant Sertraline Modulates Gene Expression and Alternative Splicing Events in the Dermatophyte Trichophyton rubrum: A Comprehensive Analysis
Source: Genes (Basel). 2025 Jan 24;16(2):146. doi: 10.3390/genes16020146 (PMC11855152; doi:10.3390/genes16020146)
Supplement: Supplementary file 1 [file genes-16-00146-s001.zip › genes-3399746-supplementary.pdf]

Supplementary material

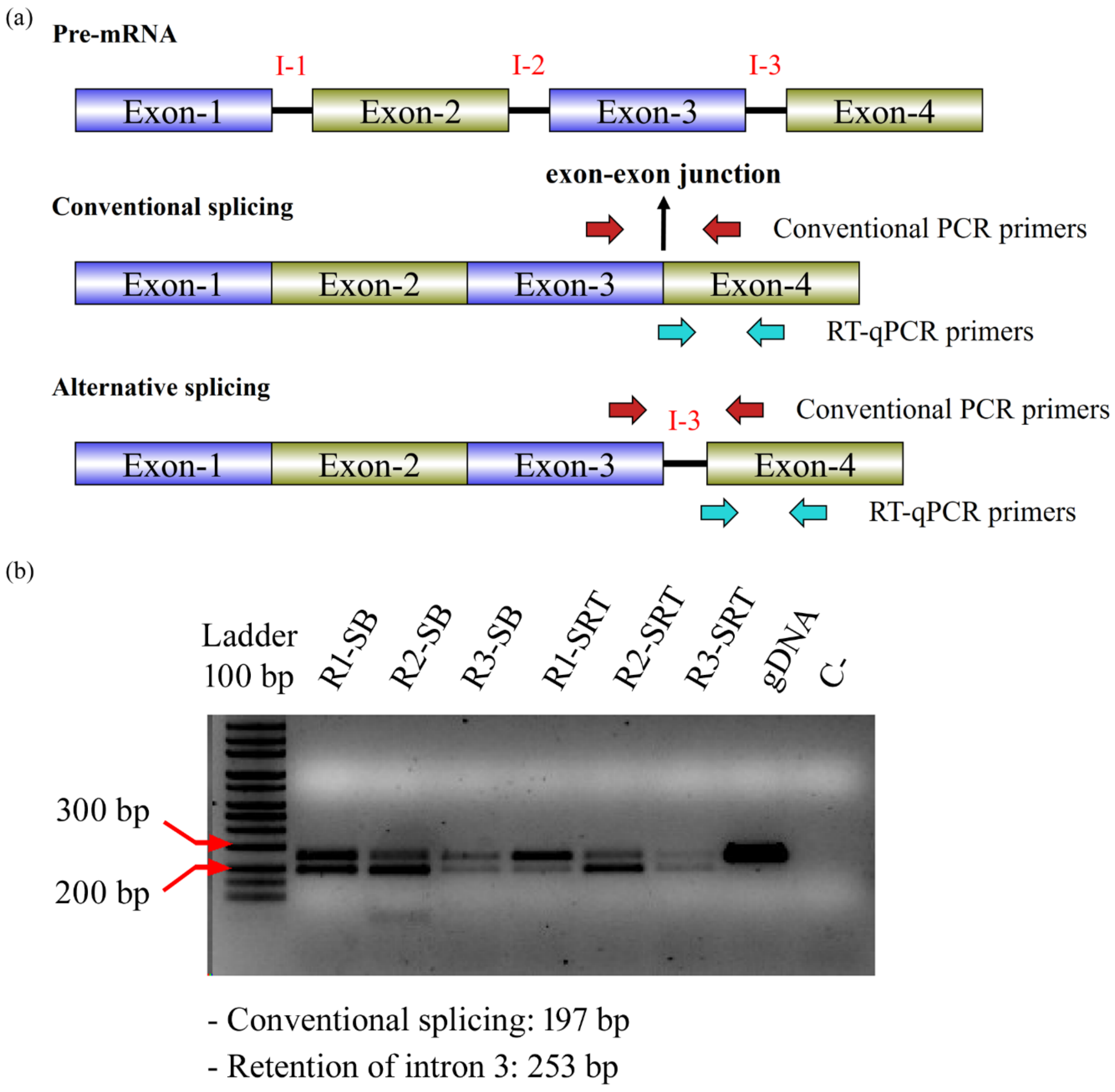

Figure S1. Validation of alternative splicing event. (a) Annealing regions of oligonucleotides used in conventional PCR and RT-qPCR assays. Splicing without retention was evaluated using primers complementary to the E-3/E-4 junction regions, while splicing with retention of intron 3 was evaluated using primers that were complementary to the I-3/E-4 junction regions. Red and green arrows indicate regions complementary to the primers. I: Intron. (b) Agarose gel with cDNA amplification products using oligonucleotides that flank intron 3 of the SRPK gene. Replicates of the control performed in Sabouraud without SRT (R1-SB, R2-SB, and R3-SB); replicates of the SRT treatment (R1-SRT, R2-SRT, and R3-SRT); Ladder: molecular weight marker (100 bp); gDNA: genomic DNA as positive control; (C-): negative control.

Table S1. Primer sets used in conventional PCR and RT-qPCR assays.

Protein/gene	ID	Primer sequences (5´- 3´)
SRPK (Conventional PCR)	TERG_07061	Fwd: GGTGGGCTTTAGAGTTAG
		Rev: CATACGCCTGACCATCCTCT
SRPK-CS (RT-qPCR)	TERG_07061	Fwd: TTACAGCCTGGGACTTGC
		Rev: GAACCGCATCATACGCCTGA
SRPK-IR (RT-qPCR)	TERG_07061	Fwd: CCTTTCATCGCTATACTGGT
		Rev: GAACCGCATCATACGCCTGA
rpb II	TERG_05742	Fwd: TGCAGGAGCTGGTGGAAGA
		Rev: GCTGGGAGGTACTGTTTGATCAA
gapdh	TERG_04402	Fwd: GCGTGACCCAGCCAACA
		Rev: CGGTGGACTCGACGATGTAGT

Table S2. List of 43 differentially expressed genes modulating alternative splicing events at 3 h and 12 h in response to sertraline. Differences were considered statistically significant at $p < 0.05$ and $|\log_2 FC| \geq 1.00$. Values in bold indicate differentially expressed genes in response to sertraline treatment. Other values indicate the differential expression of the respective introns. "I" denotes intron retention, while E(IR) indicates intron retention along adjacent exons. Red and green indicate upregulation and downregulation, respectively.

ID	Gene Product Name / AS-ID	3 h	12 h
**TERG_00010**	**amidase family protein**	**4.76**	**8.72**
	TERG_00010:I001	-1.83	-2.64
	TERG_00010:I002	-1.14	-2.09
	TERG_00010:I003	-1.29	-2.19
	TERG_00010:I004	-	-1.75
**TERG_00760**	**protein arginine methyltransferase**	**-1.71**	**-3.40**
	TERG_00760:I001	1.05	2.22
	TERG_00760:I002	-	1.58
	TERG_00760:I003	-	1.93
**TERG_00830**	**cytochrome P450 monooxygenase, putative**	**2.55**	**1.18**
	TERG_00830:I002	-	-2.50
	TERG_00830:I003	-1.88	-2.34
	TERG_00830:I004	-1.52	-1.95
	TERG_00830:I005	-	-1.32
	TERG_00830:I006	-	-1.85
**TERG_00887**	**high expression lethality protein Hel10**	**1.95**	**1.38**
	TERG_00887:I001	-1.00	-1.20
**TERG_01062**	**hypothetical protein**	**-1.78**	**-3.08**
	TERG_01062:I001	-	1.43
	TERG_01062:I002	1.05	1.94
	TERG_01062:I003	-	2.23
	TERG_01062:I004	1.40	2.27
**TERG_01572**	**carboxylesterase, putative**	**2.89**	**4.25**
	TERG_01572:I001	-1.34	-1.39
	TERG_01572:I002	-1.19	-
**TERG_01956**	**C2H2 finger domain protein, putative**	**2.12**	**3.61**
	TERG_01956:I001	-1.55	-
	TERG_01956:I002	-	-1.45
**TERG_02061**	**C2H2 transcription factor (Ace1), putative**	**1.20**	**1.89**
	TERG_02061:I001	-	-2.02
	TERG_02061:I002	-1.47	-2.92
**TERG_02186**	**ABC multidrug transporter, putative**	**1.91**	**3.47**
	TERG_02186:I002	-1.12	-2.59
**TERG_02483**	**hypothetical protein**	**1.13**	**1.99**
	TERG_02483:I001	-2.19	-1.72
**TERG_02527**	**WD40 repeat protein**	**1.29**	**1.53**
	TERG_02527:I003	-1.22	-2.45
	TERG_02527:I004	-	-2.73

**TERG_02532**	**C6 transcription factor, putative**	**2.72**	**4.90**
	TERG_02532:I001	-	-1.27
	TERG_02532:I002	-	-1.73
	TERG_02532:I003	-1.01	-1.18
**TERG_02562**	**chitin synthase C**	**1.25**	**2.38**
	TERG_02562:E003 (IR)	-1.05	-1.79
	TERG_02562:I001	-	-1.24
	TERG_02562:I003	-1.14	-
**TERG_02845**	**cercosporin toxin biosynthesis protein**	**-1.21**	**-3.00**
	TERG_02845:I002	1.63	1.66
**TERG_02878**	**eukaryotic translation initiation factor 3 subunit F**	**-1.09**	**-2.35**
	TERG_02878:E003 (IR)	1.12	2.25
**TERG_02885**	**E3 ubiquitin-protein ligase pub1**	**1.05**	**1.91**
	TERG_02885:I002	-	-1.03
	TERG_02885:I006	-1.83	-
**TERG_03078**	**cytochrome P450 oxidoreductase OrdA-like, putative**	**1.61**	**1.77**
	TERG_03078:E002 (IR)	-	-1.49
	TERG_03078:E005 (IR)	-	-1.01
	TERG_03078:E007 (IR)	-1.63	-2.10
	TERG_03078:E009 (IR)	-1.12	-2.03
	TERG_03078:I002	-1.11	-2.88
**TERG_03322**	**hypothetical protein**	**2.44**	**2.79**
	TERG_03322:I002	-1.09	-1.84
**TERG_03434**	**GTP binding protein**	**-1.10**	**-3.25**
	TERG_03434:E005 (IR)	-	1.52
	TERG_03434:I001	-1.36	-
**TERG_03662**	**hypothetical protein**	**1.31**	**2.23**
	TERG_03662:I002	-1.07	-
	TERG_03662:I004	-	-1.31
**TERG_03829**	**FAD binding domain-containing protein**	**4.86**	**6.92**
	TERG_03829:I001	-	-1.43
	TERG_03829:I002	-1.99	-2.90
**TERG_03857**	**phosphotransferase enzyme family protein**	**1.00**	**1.95**
	TERG_03857:E002 (IR)	-1.79	-1.66
	TERG_03857:E006 (IR)	-	-1.38
**TERG_03907**	**neutral amino acid permease**	**2.39**	**4.54**
	TERG_03907:I002	-	-2.01
	TERG_03907:I005	-	-1.04
	TERG_03907:I006	-1.08	-
	TERG_03907:I007	-1.08	-1.51
**TERG_04003**	**AMP dependent CoA ligase**	**2.92**	**1.25**
	TERG_04003:I001	-1.16	-1.02
	TERG_04003:I002	-1.47	-1.54
	TERG_04003:I003	-1.43	-1.40
**TERG_04224**	**ABC transporter**	**2.32**	**3.96**
	TERG_04224:I001	-1.33	-2.61

**TERG_04521**	**HypA-like protein, putative**	**2.30**	**3.15**
	TERG_04521:I001	-	-2.01
	TERG_04521:I003	-1.13	-2.06
	TERG_04521:I004	-1.38	-2.40
**TERG_04580**	**NADP-specific glutamate dehydrogenase**	**-2.64**	**-3.58**
	TERG_04580:E004 (IR)	1.25	-
	TERG_04580:E006 (IR)	1.50	1.04
	TERG_04580:E008 (IR)	1.23	-
	TERG_04580:E010 (IR)	1.57	-
	TERG_04580:I005	1.55	1.02
**TERG_04738**	**hypothetical protein**	**-1.23**	**-2.96**
	TERG_04738:I001	1.12	2.18
	TERG_04738:I004	1.09	1.44
**TERG_04769**	**extracellular serine carboxypeptidase, putative**	**1.69**	**1.85**
	TERG_04769:I001	-1.18	-
	TERG_04769:I003	-1.13	-1.11
	TERG_04769:I004	-	-1.84
**TERG_04937**	**alpha/beta hydrolase**	**5.41**	**6.40**
	TERG_04937:I001	-1.68	-1.62
	TERG_04937:I002	-1.77	-1.32
**TERG_05023**	**calcium/proton exchanger**	**3.86**	**2.95**
	TERG_05023:I001	-1.54	-
	TERG_05023:I004	-1.13	-1.07
**TERG_05303**	**phospholipase PldA, putative**	**1.50**	**1.88**
	TERG_05303:E003 (IR)	-1.91	-2.15
	TERG_05303:I001	-	-1.00
**TERG_05435**	**hypothetical protein**	**-1.70**	**-1.62**
	TERG_05435:I001	-	-1.10
	TERG_05435:I002	-1.11	-
	TERG_05435:I003	-1.54	-
**TERG_05585**	**short-chain dehydrogenase/reductase SDR**	**-1.94**	**-2.99**
	TERG_05585:I001	-	1.72
	TERG_05585:I003	1.09	1.49
**TERG_06089**	**PH domain-containing protein**	**1.38**	**1.74**
	TERG_06089:E002 (IR)	-1.29	-2.87
	TERG_06089:E004 (IR)	-1.67	-3.33
	TERG_06089:I003	-	-2.10
**TERG_06116**	**indoleamine 2,3-dioxygenase**	**2.69**	**1.02**
	TERG_06116:E004 (IR)	-1.05	-1.50
	TERG_06116:I002	-	-1.12
	TERG_06116:I004	-	-1.72
**TERG_06145**	**arrestin**	**1.19**	**2.02**
	TERG_06145:I002	-1.11	-
	TERG_06145:I003	-	-1.23
**TERG_06315**	**integral membrane protein**	**-1.08**	**-2.44**
	TERG_06315:I001	-	-2.32

	TERG_06315:I006	1.33	-
**TERG_06347**	**hypothetical protein**	**4.53**	**5.85**
	TERG_06347:E003 (IR)	-2.98	-4.73
	TERG_06347:E007 (IR)	-1.16	-1.41
	TERG_06347:I004	-1.42	-1.16
**TERG_06358**	**dicer**	**-1.14**	**-1.68**
	TERG_06358:I001	1.22	2.03
	TERG_06358:I002	1.33	1.80
	TERG_06358:I005	-1.69	-
	TERG_06358:I007	2.04	-
**TERG_07095**	**hypothetical protein**	**1.55**	**1.90**
	TERG_07095:I002	-1.00	-1.32
**TERG_07200**	**C2 domain-containing protein**	**-1.78**	**-2.48**
	TERG_07200:I001	1.11	-
	TERG_07200:I003	1.91	1.23
	TERG_07200:I004	1.51	1.22
**TERG_07786**	**NAP family protein**	**-1.35**	**-2.55**
	TERG_07786:I001	-	1.40
	TERG_07786:I002	1.12	1.75

A *p*-value < .05 and |log2FC| ≥ 1.00 is considered statistically significant.
Abbreviations: AS, alternative splicing; ID, identification; h, hours.

Table S3. Alternative splicing events in the pre-mRNA of kinase genes in response to sertraline. The differential expression of these genes is indicated.

Gene-ID	Gene Product Name	DEG		AS-ID	AS	
		3h	12h		3h	12h
TERG_00412	CMGC/SRPK protein kinase	-	-	TERG_00412:I001	-2.07	-
TERG_00428	phosphotransferase enzyme family protein	-	-	TERG_00428:I002	-1.01	-1.12
TERG_00689	AUR protein kinase	-1.16	-3.75	TERG_00689:I001	-	1.37
				TERG_00689:I002	-	2.24
TERG_00694	glutamate 5-kinase	-1.77	-3.65	TERG_00694:E005 (IR)	-	1.19
				TERG_00694:I001	-	1.05
				TERG_00694:I002	-	1.86
TERG_00768	serine/threonine protein kinase	-	-	TERG_00768:I001	-	-1.27
				TERG_00768:I005	-	-1.41
TERG_00875	glutamate-cysteine ligase	1.76	-	TERG_00875:E004 (IR)	-	-1.04
				TERG_00875:E008 (IR)	-	-1.04
				TERG_00875:I006	-1.07	
TERG_00915	CAMK protein kinase	-	-	TERG_00915:I003	-	1.49
TERG_00978	RNA processing protein Grc3, putative	-	-	TERG_00978:E003 (IR)	-	1.12
				TERG_00978:E007 (IR)	-	1.18
TERG_01071	phosphotransferase	-	-	TERG_01071:I001	-	1.55
				TERG_01071:I002	-	1.52
TERG_01333	AGC/NDR/NDR-UNCLASSIFIED protein kinase	-	-1.17	TERG_01333:I002	-	-1.43
TERG_01475	DNA topoisomerase 2	-	-	TERG_01475:I003	-	-2.59
TERG_01761	hypothetical protein	-	-1.54	TERG_01761:I001	-	-1.42
TERG_02018	hypothetical protein	1.27	2.02	TERG_02018:I004	-1.08	-
TERG_02309	serine/threonine protein kinase	-	-	TERG_02309:I002	-	1.11
TERG_02677	CDC7 protein kinase	-	-1.17	TERG_02677:I002	1.22	-
				TERG_02677:I003	1.22	-
TERG_03017	AGC protein kinase	-	-	TERG_03017:E005 (IR)	-	-1.79
				TERG_03017:I003	-	-1.98
TERG_03379	AGC/NDR/NDR protein kinase	-	-	TERG_03379:I002	-	2.31
TERG_03876	serine/threonine protein kinase	-	-	TERG_03876:I001	-	-2.87
TERG_03940	hypothetical protein	-	-	TERG_03940:I001	-1.02	-
TERG_03991	phosphotransferase enzyme family protein	-	-	TERG_03991:I003	-	1.07
TERG_04058	STE/STE11 protein kinase	-	-	TERG_04058:I001	-	-1.31
TERG_04107	FunK1 protein kinase	-	-	TERG_04107:I001	-	-2.48
TERG_04247	CMGC/SRPK protein kinase	-	-	TERG_04247:I003	-1.14	-
TERG_04374	STE/STE20/YSK protein kinase	-1.34	-3.18	TERG_04374:I005	-	-2.27
TERG_04395	phosphotransferase enzyme family protein	-	-	TERG_04395:I003	-	-1.29
TERG_04429	MORN repeat-containing protein	-	1.84	TERG_04429:I001		-2.06
TERG_04558	nucleoside diphosphate kinase	-1.57	-2.48	TERG_04558:I001	-	1.68
				TERG_04558:I003	-	2.11
				TERG_04558:I004	-	1.47

TERG_05107	AGC/RSK/RSK-UNCLASSIFIED protein kinase	-	-	TERG_05107:I001	-	1.13
TERG_05197	protein kinase activator	-	-	TERG_05197:I003	-	-1.41
				TERG_05197:I004	-	-1.22
TERG_05380	protein kinase regulator Ste50	-	-	TERG_05380:E004 (IR)	-	-2.42
TERG_05462	CMGC/SRPK protein kinase	-	-	TERG_05462:I003	-	1.30
TERG_05552	DNA mismatch repair protein MutL	1.14	1.53	TERG_05552:I001	-	-1.06
TERG_05821	pyruvate kinase	-	1.10	TERG_05821:E003 (IR)	-	-3.01
				TERG_05821:I002	-	-3.11
				TERG_05821:I003	-	-1.79
				TERG_05821:I004	-	-2.21
				TERG_05821:I005	-	-1.73
				TERG_05821:I006	-	-1.32
TERG_06151	pyridoxine kinase	1.15	1.15	TERG_06151:I001	-	-1.73
				TERG_06151:I002	-	-1.62
				TERG_06151:I003	-	-1.00
				TERG_06151:I004	-	-1.17
				TERG_06151:I005	-	-1.74
TERG_06340	AGC/PKC protein kinase	-	-	TERG_06340:E004 (IR)	-1.32	
TERG_06403	HASPIN protein kinase	-1.12	-2.97	TERG_06403:I001	-	-3.27
TERG_06422	6-phosphofructo-2-kinase/fructose-2,6-bisphosphatase	-	-	TERG_06422:I001	-	-1.12
TERG_06789	CDK-activating kinase assembly factor MAT1	-	-	TERG_06789:E002 (IR)	1.08	-
TERG_07058	Hsp70 family chaperone, putative	1.24	1.42	TERG_07058:E003 (IR)	-	-1.93
				TERG_07058:E005 (IR)	-	-2.31
**TERG_07061**	**CMGC/SRPK protein kinase**	**-**	**-1.61**	**TERG_07061:I003**	**1.43**	**2.50**
TERG_07563	DNA 3'-phosphatase	-	-	TERG_07563:I001	-	1.12
TERG_07751	serine/threonine protein kinase	-	-	TERG_07751:I001	-	-1.35
TERG_07791	hypothetical protein	-	-	TERG_07791:I001	1.26	-
TERG_08064	choline kinase, putative	-	-	TERG_08064:I001	-	-1.19
TERG_08136	CMGC protein kinase	-	-1.14	TERG_08136:I003	-1.01	-
TERG_08189	CMGC/CLK protein kinase	-	-	TERG_08189:I004	-1.00	-3.99
TERG_08251	CMGC/SRPK protein kinase	-	-	TERG_08251:I003	1.36	-
TERG_08319	CMGC/SRPK protein kinase	-	-	TERG_08319:I002	-1.25	-
TERG_08452	phosphotidylinositol kinase Tel1, putative	-	-	TERG_08452:I005	-	-1.19
TERG_08455	dihydroxyacetone kinase	-	-1.34	TERG_08455:I001	-	-1.24
TERG_08794	pyruvate dehydrogenase kinase	-	-1.94	TERG_08794:I001	-	-1.15
TERG_11628	glutamine synthetase	-	-	TERG_11628:I002	-	-1.37
				TERG_11628:I004	-	-1.44
TERG_11690	protein kinase subdomain-containing protein	-1.35	-1.86	TERG_11690:I003	-1.09	-
				TERG_11690:I004	-1.26	-

A *p*-value < .05 and |log2FC| ≥ 1.00 is considered statistically significant. Highlighting the gene that encodes the SRPK protein kinase.
Abbreviations: AS, alternative splicing; DEG, differential expressed genes; ID, identification; h, hours.
